# Volumetric MR-Guided High-Intensity Focused Ultrasound with Direct Skin Cooling for the Treatment of Symptomatic Uterine Fibroids: Proof-of-Concept Study

**DOI:** 10.1155/2015/684250

**Published:** 2015-08-30

**Authors:** Marlijne E. Ikink, Johanna M. M. van Breugel, Gerald Schubert, Robbert J. Nijenhuis, Lambertus W. Bartels, Chrit T. W. Moonen, Maurice A. A. J. van den Bosch

**Affiliations:** ^1^Department of Radiology, University Medical Center Utrecht, Heidelberglaan 100, 3584 CX Utrecht, The Netherlands; ^2^Philips Healthcare, Philips Medical Systems MR, Äyritie 4, 01511 Vantaa, Finland; ^3^Department of Radiology and Image Sciences Institute, University Medical Center Utrecht, Heidelberglaan 100, 3584 CX Utrecht, The Netherlands

## Abstract

*Objective.* To prospectively assess the safety and technical feasibility of volumetric magnetic resonance-guided high-intensity focused ultrasound (MR-HIFU) ablation with direct skin cooling (DISC) during treatment of uterine fibroids.* Methods.* In this proof-of-concept study, eight patients were consecutively selected for clinical MR-HIFU ablation of uterine fibroids with the use of an additional DISC device to maintain a constant temperature (*T* ≈ 20°C) at the interface between the HIFU table top and the skin. Technical feasibility was verified by successful completion of MR-HIFU ablation. Contrast-enhanced T1-weighted MRI was used to measure the treatment effect (nonperfused volume (NPV) ratio). Safety was evaluated by recording of adverse events (AEs) within 30 days' follow-up.* Results.* All MR-HIFU treatments were successfully completed in an outpatient setting. The median NPV ratio was 0.56 (IQR [0.27–0.72]). Immediately after treatment, two patients experienced coldness related discomfort which resolved at the same day. No serious (device-related) AEs were reported. Specifically, no skin burns, cold injuries, or subcutaneous edema were observed.* Conclusion.* This study showed that it is safe and technically feasible to complete a volumetric MR-HIFU ablation with DISC. This technique may reduce the risk of thermal injury to the abdominal wall during MR-HIFU ablation of uterine fibroids. This trial is registered with NTR4189.

## 1. Introduction

Over the last decade, minimally or noninvasive treatment options have gained popularity and continue to evolve and expand with developments in technology and with growing experience. Numerous technological advances have been driven by the observed benefits of the minimally invasive approach, including less side effects, shorter recovery time, and favorable cosmetic results. Since the first feasibility report in 2003 [1], magnetic resonance-guided high-intensity focused ultrasound (MR-HIFU) has been successfully employed to treat symptomatic uterine fibroids in a clinical setting. Although not all uterine fibroids are symptomatic, they are in at least 25% of the Caucasian women in their reproductive years associated with significant morbidity, including abnormal menstrual bleeding, pelvic discomfort, and reproductive dysfunction [2, 3]. An increasing number of symptomatic patients demand less invasive treatment methods in order to achieve symptom relief and a better quality of life. MR-HIFU offers the advantage to perform completely noninvasive thermal ablation because the ultrasound transducer is located outside the abdomen and steers high-intensity focused ultrasound energy into the targeted area through the intact skin.

Since 2010, a volumetric MR-HIFU system has been available for routine clinical treatments of uterine fibroids [4]. The volumetric ablation approach utilizes the accumulation of heat by electronically steering the focus along outward-moving concentric circles, producing well-defined regions of protein denaturation, irreversible cell damage, and coagulative necrosis. However, the treatment of larger ablation volumes requires more thermal energy which may lead to a temperature increase along the ultrasound beam axis in the near field (i.e., intermediate layers located between the ultrasound transducer and the target region, such as epidermis, dermis, subcutaneous tissue, and deeper abdominal layers) [5]. During periods of thermal stress, the rate of heat transfer through the skin surface depends primarily on the heat capacity (or ability to absorb heat) and the thermal conductivity (or ability to transfer heat) of the skin to facilitate heat loss [6]. This heat flux may be enhanced through blood circulation by carrying the heat to adjacent tissues [7, 8]. Temperature rise within the skin layers will mainly occur in the subcutaneous tissue due to its lower specific heat capacity [6], insulator properties [6, 7, 9], and its lower blood supply [10–12] than that of other tissues in the abdominal wall. Additionally, reflections at boundaries between different media (e.g., air-skin and/or skin-fat) can occur because of differences in the acoustic impedance of various media [13]. The transmission losses from reflection at the skin interface and attenuation through the skin layers might lead to hot spots and skin overheating. Although MR-HIFU ablation of uterine fibroids is related to a low complication rate, skin toxicity and abdominal discomfort have been described by several groups [1, 14–21]. Undesired heat accumulation in the near field and target area is moderated by enforcing conservative cooling times between the subsequent energy depositions (sonications) to prevent irreversible thermal tissue damage [22, 23], such as skin burns, subcutaneous edema formation, or fat necrosis [24]. The cooling time is chosen to ensure return of the heated tissue layers to body temperature, and cooling ranges typically a few minutes per energy delivery. This leads to undesirably long delays between the sonications, which contributes to prolonged overall treatment times. It would therefore be valuable to regulate the temperature of the skin layers at a constant (room) temperature to reduce thermal tissue damage during MR-HIFU ablation, and accordingly, speed up the treatment procedure.

In this study we demonstrate the concept for the clinical use of a direct skin cooling (DISC) device added to an MR-HIFU system during volumetric ablation of uterine fibroids. The purpose of this study was to evaluate the feasibility and safety of uterine fibroid treatments using this DISC system as additional buffer against potential adverse events related to skin heating.

## 2. Materials and Methods

### 2.1. Patients and Lesions

This prospective nonrandomized proof-of-concept study (NL45458.041.13) was approved by the Institutional Review Board and was conducted in accordance with the rules for international good clinical practice. Patients who participated in this study were already selected for clinical MR-HIFU ablation of uterine fibroids based on their history, physical examination, and diagnostic pelvic MRI examination. Routine inclusion and exclusion were carried out; eligible patients met the following inclusion criteria [21]: (1) 18 years or older, (2) clinically diagnosed with symptomatic uterine fibroids, (3) referred by their gynecologists with an absolute indication for intervention, (4) premenopausal or perimenopausal, (5) not currently pregnant or breastfeeding, (6) no general contraindications for magnetic resonance imaging (MRI) and MR contrast agents, and (7) able to undergo the MR-HIFU procedure based on a diagnostic pelvic MRI examination in prone position (Achieva 1.5-T, Philips Healthcare, Best, The Netherlands). Exclusion criteria were (1) the presence of other pelvic diseases, (2) unavoidable extensive scar tissue in the lower abdomen (in some cases alternate ultrasound beam paths were possible to avoid scar tissue, e.g., via beam shaping or beam angulation), (3) interposition of the bowel between the anterior abdominal wall and the dominant uterine fibroid, (4) excessive fibroid size (≥12 cm), and (5) too many lesions (≥10 uterine fibroids). All patients gave written informed consent for conducting an MR-HIFU procedure with the presence of the direct skin cooling (DISC) device.

### 2.2. MR-HIFU System

**Figure 1:**
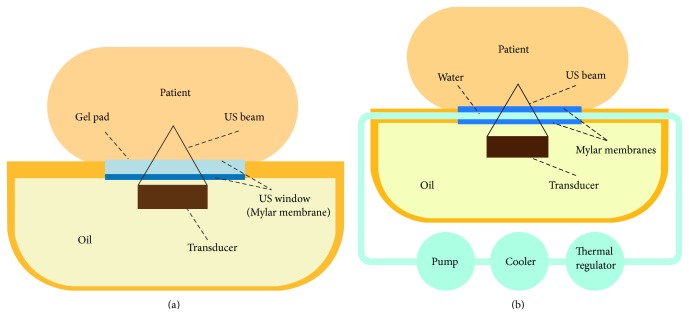
Schematic illustration of the differences between the clinical volumetric MR-HIFU system with (b) and without (a) the presence of the investigational DISC device.

All treatments were performed on a modified clinical MR-HIFU fibroid therapy system (Sonalleve, Philips Healthcare, Vantaa, Finland) integrated into a 1.5-T MRI scanner (Achieva, Philips Healthcare, Best, The Netherlands). MR images were used for treatment localization, feedback control (beam guidance), real-time temperature mapping with the proton resonance frequency shift (PRFS) thermometry method, and posttreatment verification of the ablated tissue. The curved patient MR table top incorporated a phased-array 256-channel HIFU transducer (radius of curvature: 14 cm, operating at 1.2 MHz) housed in an electromechanical positioning system to deliver spatially and temporally controlled heating. The DISC device consisted of a liquid (water) cooling reservoir which was mounted on top of the standard clinical MR-HIFU table top, between the degassed liquid (oil) bath—in which the HIFU transducer is immersed—and the patients' skin. A schematic illustration of the clinical volumetric MR-HIFU system with and without the presence of the investigational DISC device is shown in Figure 1. The water cooling reservoir was connected to a water pump, cooling element, temperature regulator, degasser, and a bubble-trap to assure that air bubbles were extracted from the DISC system. The temperature of the water cooling reservoir was regulated at a constant room temperature (*T* ≈ 20°C), such that the temperature was well tolerated on bare skin. The DISC system was filled with degassed water and turned on (10 minutes) before the start of the MR-HIFU treatment to establish the target temperature of the water cooling reservoir. By the active displacement of water through the liquid cooling reservoir (circuit) a stable temperature could be guaranteed. The temperatures in the water cooling reservoir were measured using a fiber-optical temperature sensor (SoftSens, Opsens Inc., Québec, QC, Canada), which was placed in the water between the two Mylar (polyethylene terephthalate) membranes outside the immediate acoustic beam path. Since the skin is in direct contact with the cooled water reservoir (separated only by a 50 *μ*m thick membrane), the measured water temperature directly reflects the patient's skin temperature throughout the MR-HIFU ablation. Local hotspots on skin level that appear during individual sonications are equilibrated on timescales of some tens of seconds. During acquisition of diagnostic MR images, the flow within the water cooling reservoir was stopped to prevent artifacts on the MR data.

### 2.3. MR-HIFU Procedure

**Figure 2:**
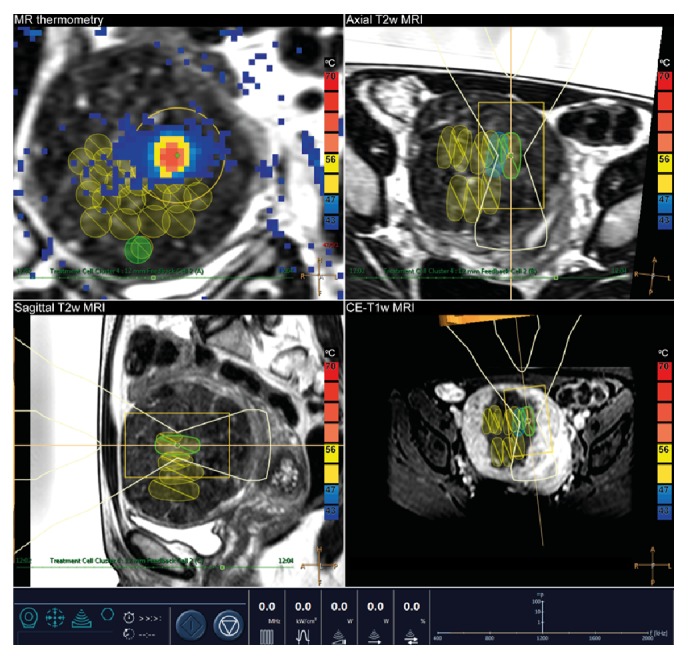
Typical representation of the MR images in the MR-HIFU user interface during treatment of uterine fibroids. The patient was lying in prone position (feet first) on the MR table top with the integrated direct skin cooling (DISC) device. The uterine fibroid was positioned directly above the MR-HIFU transducer. The ultrasound beam path was planned using T2-weighted MRI in three orthogonal planes, that is, coronal (top left), axial (top right), and sagittal (bottom left) plane. During each sonication, color temperature maps were computed by the MR-HIFU system using the proton resonance frequency shift (PRFS) thermometry method and shown on top of the anatomical images (top left). Immediately after MR-HIFU treatment, the volume that was successfully treated was defined as the nonenhancing part of the fibroid on contrast-enhanced T1-weighted MRI (bottom right).

All patients were treated in an outpatient setting. Patient preparation on the day of the MR-HIFU procedure (i.e., hair removal of the lower abdomen; insertion of an intravenous line and Foley catheter), treatment planning, and the volumetric ablation protocol have been described in previous publications [19, 21]. Cooling times were enforced as in normal MR-HIFU treatments, so that the DISC device was used as an additional safety buffer against potential adverse events related to skin heating. The required cooling times of at least 90 seconds were respected as suggested by the feedback MR-HIFU system. A standardized preprocedural pain management protocol was used with paracetamol 1,000 mg intravenous (Paracetamol Kabi, 10 mg/mL, Fresenius Kabi Nederland B.V., Schelle, Belgium), diclofenac 75 mg intravenous (Voltaren, 25 mg/mL, Novartis Pharma B.V., Arnhem, The Netherlands), and oxycodone 5 mg capsules (OxyNorm, 5 mg, Mundipharma Pharmaceuticals B.V., Hoevelaken, The Netherlands). In this study, patients were asked to lie down in prone position (feet first) on the patient MR table top with the integrated DISC device. A wetted ultrasound gel pad (7.5 mm) or a thin mixture (10 : 1) of degassed water and ultrasound gel (gel film) was used as coupling agents to provide adequate direct contact for the ultrasound waves to penetrate the patients' skin. Two different types of acoustic couplers were evaluated, in order to assess whether treatment could also be carried out without the commonly used gel pad. Standard MR images were acquired to detect any obstacles in the ultrasound beam path and the contact surface to ensure that MR-HIFU ablation was safe with respect to heating in unwanted locations due to the presence of air bubbles, scars, bowel, bone, and/or implants. A typical representation of the MR images in the MR-HIFU user interface is shown in Figure 2. The following MR sequences were used: coronal membrane bubble scan (three-dimensional (3D) spoiled gradient echo (FFE) with repetition time [TR], 5.8 milliseconds [ms]; echo time [TE], 4.0 ms; flip angle [FA], 6°; field of view [FOV], 260 mm × 260 mm; acquired [ACQ] voxel size, 1.00 × 1.00 × 2.00 mm^3^; reconstructed [REC] voxel size, 0.49 × 0.49 × 1.00 mm^3^; number of averages [NSA], 6; acquisition time, 00:39 minutes), coronal skin bubble scan (multislice single-echo FFE with TR, 150 ms; TE, 15 ms; FA, 55°; FOV, 280 mm × 280 mm; ACQ voxel size, 1.25 × 1.25 × 2.50 mm^3^; REC voxel size, 0.31 × 0.31 × 2.50 mm^3^; NSA, 2; acquisition time, 00:38 minutes), coronal scar scan (single-echo 3D FFE with TR, 21 ms; TE, 6.0 ms; FA, 15°; FOV, 200 mm × 200 mm; ACQ voxel size, 0.89 × 0.89 × 2.00 mm^3^; REC voxel size, 0.31 × 0.31 × 1.00 mm^3^; NSA, 3; acquisition time, 02:06.5 minutes), and anatomical 3D T2-weighted (T2w) turbo spin echo (TSE) with TR, 1425 ms; TE, 130 ms; FA, 90°; FOV, 250 mm × 250 mm; ACQ voxel size, 1.20 × 1.39 × 3.00 mm^3^; REC voxel size, 0.49 × 0.49 × 1.50 mm^3^; NSA, 2; acquisition time, 03:50.8 minutes, and 3D T1-weighted (T1w) FFE with TR, 3.6 ms; TE, 1.90 ms; FA, 7°; FOV, 220 mm × 240 mm; ACQ voxel size, 1.25 × 1.53 × 2.50 mm^3^; REC voxel size, 0.47 × 0.47 × 1.25 mm^3^; NSA, 8; acquisition time, 01:57.3 minutes. During the treatment procedure, patients received conscious sedation (propofol-ketamine combination) to reduce and control pain or discomfort and involuntary movements. Monitored anesthesia care (MAC) was provided by the anesthesiologist (procedural sedation specialist), which included preprocedural screening, intraprocedural support of vital functions and administration of anesthetic agents, and postprocedural anesthesia management. After completion of the MR-HIFU procedure, a set of MR images of the target region was obtained including a contrast-enhanced (gadobutrol, Gadovist, 0.1 mmol/kg, Bayer Schering Pharma) T1-weighted (CE-T1w) TFE sequence (with TR, 5.4 ms; TE, 2.6 ms; FA, 10°; FOV, 250 mm × 250 mm; ACQ voxel size, 1.49 × 1.89 × 3.00 mm^3^; REC voxel size, 0.49 × 0.49 × 1.50 mm^3^; NSA, 4; acquisition time, 02:14.5 minutes) to allow a sum-of-slice measurement of the nonperfused volume (NPV), indicating the volume of fibroid tissue that is nonviable. Following this, patients were conducted to the recovery room for medical supervision before being discharged from the hospital on the same day.

### 2.4. Data Collection

The primary endpoint of this study was the technical feasibility of clinical MR-HIFU fibroid treatments with direct skin cooling, as determined by recording the successfully completed treatments using the investigational DISC device. Treatment completion was judged by an experienced operating physician (M.v.d.B.). If a treatment was aborted before the desired ablation volume was achieved and the backup CE-labelled MR-HIFU system had to be used, the treatment was counted as a failure. The extent of treatment was reported by measuring the NPV ratios, defined as the nonenhancing part of the fibroid divided by the fibroid volume. The achieved NPV ratios were compared to data from the literature to assess whether the performed treatments represent typical MR-HIFU ablations. In order to determine the effectiveness of the MR-HIFU treatments with the DISC device, the energy deposition rate (in kilojoule per hour [kJ/h]) was calculated by dividing the deposited treatment energy [kJ] by the total treatment time (1/[h]). The treatment time was defined as the time from the start of the first to the end of the last sonication.

**Figure 3:**
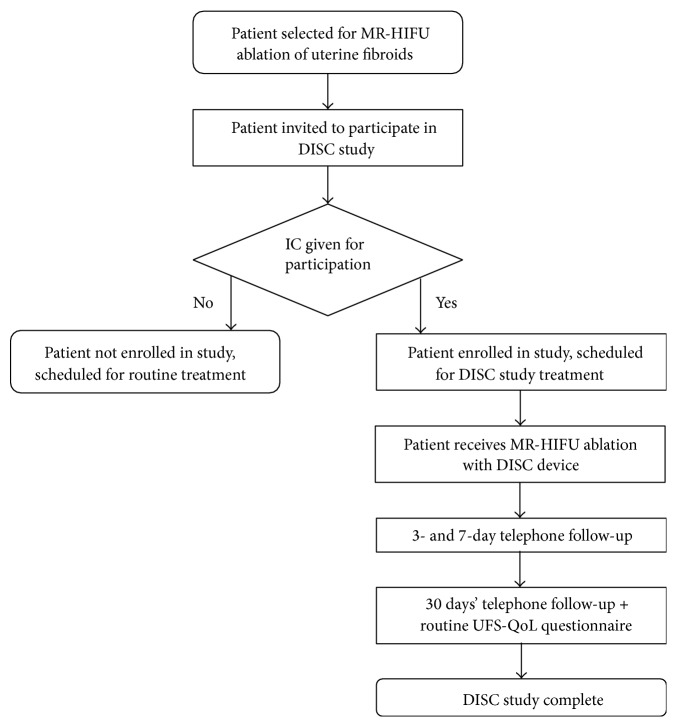
Flowchart shows the patients' progress (*n* = 8) through the DISC study. MR-HIFU: magnetic resonance-guided high-intensity focused ultrasound; DISC: direct skin cooling; IC: informed consent; UFS-QoL: uterine fibroid symptom and health-related quality of life.

The secondary endpoint of this study was to gain insights into factors influencing the safety. Safety was assessed by recording (serious) adverse events and whether they were related to the investigational DISC device, in particular by inspection of patients' skin immediately after MR-HIFU treatment. All adverse events (AEs) were recorded and classified following the 14155:2011 standard for Good Clinical Practice in clinical investigation of medical devices for human subjects issued by the International Organization for Standardization (ISO). Any AEs observed during or after MR-HIFU treatment were followed and documented until they had abated or until a stable situation had been reached. Patients were contacted by telephone 3 days, 7 days, and 30 days after the MR-HIFU procedure to determine whether any AEs had occurred. A pain assessment scale was obtained using the visual analogue scale (VAS) from 0 (no pain) to 10 (worst pain possible). The flow chart in Figure 3 shows the patients' progress through the study.

### 2.5. Statistical Analysis

Data were prospectively collected and analyzed to evaluate the safety and feasibility of performing MR-HIFU treatment of uterine fibroids in a volumetric MR-HIFU system equipped with a DISC device. The sample size calculation was based on the two-sided Agresti-Coull 95% confidence interval (95% CI) [25]. The Agresti-Coull interval is known to provide optimal coverage for binominal proportions when the sample size is small and the success probability approaches 0 or 1 [26, 27]. The estimate of the Agresti-Coull 95% CI showed that 8 successfully treated patients would be needed to detect a therapy success rate of at least 63%. Estimations for different proportions of successful therapy completion are presented in Table 1. Descriptive statistics were used to describe the distribution of the patients' demographic and lesion characteristics. The treatment data of each patient was subsequently summarized and reported. Categorical data are presented in number and percentage, whereas continuous data are given in median and interquartile range (IQR). Statistical analyses were performed using IBM SPSS Statistics, version 20.0 (Armonk, New York, USA).

## 3. Results

Eight Caucasian patients with nine treatable uterine fibroids were consecutively enrolled in this study. One patient was treated for two uterine fibroids during the same MR-HIFU treatment. Three patients had a scar in the lower abdomen: one patient (ID number 4) had undergone an abdominal myomectomy (Pfannenstiel incision), one patient (ID number 1) underwent an open appendectomy (McBurney incision), and one patient (ID number 2) had minor laparoscopic scars after a diagnostic laparoscopy due to chronic abdominal pain. In patient 4, urinary bladder filling (with a saline solution) was used to avoid the surgical scar in the ultrasound beam path. Baseline data collected for each patient at the beginning of the study are presented in Table 2. All MR-HIFU treatments were successfully completed using the investigational DISC device; no technical failure was observed. The use of the backup CE-labeled MR-HIFU system was not necessary. The median treatment time was 192 minutes (IQR [180–225]), with a median energy deposition rate of 67 kJ/h (IQR [55–92]). The median volume of the uterine fibroids was 147 cm^3^ (IQR [49–338]); the median maximum fibroid diameter was 7.7 cm (IQR [5.1–8.8]). A median nonperfused volume of 56 cm^3^ (IQR [14–91]) was achieved, and a median NPV ratio of 0.56 (IQR [0.27–0.72]) was found. In one patient (ID number 7), no NPV could be achieved due to insufficient heating probably as a result of the tissue characteristics of the uterine fibroid (type 3) [28].  Table 3 shows an overview of the treatment data of each patient treated in this study.

No serious (device-related) adverse events were observed within 30 days' follow-up. No patient required prolonged observation before discharge or readmission after hospital discharge. In particular, no skin burns, cold injuries, or subcutaneous edema (determined by increased signal intensity on T2w images) was observed in patients treated with the DISC device. Several mild AEs were reported, such as abdominal pain or cramping (*n* = 3), back pain (*n* = 3), abdominal tenderness (*n* = 2), ergonomic problems (*n* = 2), namely, fibular compression neuropathy due to external pressure at the right fibular head during a long treatment procedure of 230 minutes (ID number 3) and pressure marks due to the curved patient table top, dyspepsia and constipation (*n* = 2), dizziness (*n* = 3), and lethargy (*n* = 4). One mild device-related AE occurred, namely, coldness-related discomfort which was rapidly resolved with a hot water bottle on the day of treatment (*n* = 2). At the moment of hospital discharge, the median VAS score was 1.5 (IQR [0.25–3.0], range [0–7]). Typically, AEs resolved within 7 days' follow-up and patients were able to resume their daily activities again. However, after 30 days, two patients were still recovering from ongoing AEs (constipation and lethargy), both probably related to the administration of anaesthetic agents.

## 4. Discussion

During MR-HIFU treatments, undesired heating outside of the targeted ablation area may occur, in particular in the near field region of the HIFU beam. Currently, the risk of near field damage is mitigated by the time-consuming enforcement of cooling periods between ultrasound sonications. Introduction of a cooled interface which allows direct cooling of the patients' skin may further mitigate undesired heating (by shifting the baseline temperature), potentially increasing treatment efficacy and providing an additional buffer. In this proof-of-concept study we have demonstrated the safety and feasibility of using a direct skin cooling (DISC) device added to a volumetric MR-HIFU system for uterine fibroid treatments. To the best of our knowledge, this concept had not yet been investigated in a clinical setting. Our results showed that it is technically feasible and safe to complete an MR-HIFU treatment with a DISC device. No thermal damage to the near field, due to temperature increase of the ultrasound energy emitted from the HIFU transducer, was observed in this study. All eight patients (100%) could be treated as in normal clinical practice, representing a typical case series of uterine fibroid MR-HIFU treatments. No clinical or technical problems have occurred preventing MR-HIFU treatments using the DISC device to be successfully completed. No serious (device-related) AEs occurred after treatment and the achieved median NPV ratio (0.56 (IQR [0.27–0.72]) was comparable to data previously published. Reported NPV ratios ranged from 0.40 to 0.70 with a median NPV ratio of 0.56 [21, 29–33]. Specifically, no skin redness and/or irritation of the abdominal wall—either related to skin heating or skin cooling—were seen. As known, cutaneous microvascular reactivity (namely, thermoregulatory reflex) is essential to maintain the human core temperature during challenges to thermal hemostasis [34, 35]. Prolonged exposure of direct cooling of the skin will eventually lead to cutaneous vasoconstriction [36–38], which may induce ischemia. Extended exposure to localized cold-induced vasoconstriction may cause various injuries to the patients' skin that typically fall within the domain termed nonfreezing cold injury (NFCI) [39, 40]. However, these injuries (e.g., chilblains or immersion foot) are most commonly reported to the body's lower extremities, such as the legs and feet [41, 42]. In addition, the literature evidence indicates that no serious thermal tissue damage is to be expected within several hours until freezing occurs [43, 44]. In general, NFCI occurs at temperatures of 0°C to 15°C. Considering the temperatures and exposure times relevant for patients during an MR-HIFU procedure, it can be concluded that the probability of occurrence for this specific low temperature event was indeed very unlikely.

It should be noted that this is a technical feasibility study with a small number of patients (*n* = 8). Despite the promising preliminary results, the current study was not designed to measure the efficacy of the cooling system. Therefore, further research is needed to assess the clinical efficacy of this investigational DISC device as add-on to the volumetric MR-HIFU system. In a future step, a clinical follow-up study will investigate to which extent the DISC system allows for speeding up uterine fibroid MR-HIFU treatments by benefiting from reduced cooling times. After each sonication, the cooling effect in the subcutaneous tissue layers will be systematically monitored using the T2-based thermometry as recently described by Baron et al. [45]. The acquired temperature (cool-down) information in the near field can be used to provide thermal feedback to the MR-HIFU system and use this to adjust the cooling periods between the subsequent energy depositions in real time. This novel technique may result in some advantages for the patient and their treating physician: (1) ablation of larger volumes of (fibroid) tissue in the shortest period of time that is safely possible and (2) more efficient treatment of type 3 uterine fibroids with a high signal intensity on T2w imaging [28, 46]. The fact that it is technically feasible to produce thermal lesions safely in the present study highlights the potential of this DISC system in the future MR-HIFU ablations of uterine fibroids. Another practical advantage of using DISC is that it seems sufficient to use a gel film (water-gel mixture) for acoustic coupling between the table top membrane and the depilated skin of the patient. Previously, it was easy to trap air bubbles between the commonly used (15 mm) gel pad and the patients' skin in the process of patient positioning. Since air bubbles may reflect the ultrasound energy—due to their low acoustic impedance [47]—they can cause abdominal pain or discomfort and skin burns. This restriction appears alleviated now by the use of a gel film, because we did not observe any air bubbles after positioning. Furthermore, without the insulating gel pad, the patient profits directly from the cooling effect without dissipation. Finally, the DISC system with gel-film has the same thickness as the old gel pads (Figure 1). This means that it is possible to reach targets as deep in the patient body as before. Consequently, the use of a gel film will potentially reduce risks and offers opportunities to speed up the patient preparation and overall treatment time.

Several limitations to the present study need to be acknowledged. First, we did not analyze the patient-reported outcomes of symptom severity (tSSS) and health-related quality of life (HRQoL) as measure of the treatment effect. The focus of the current study was to verify that the use of the DISC device did not affect the performance of the volumetric MR-HIFU system. The upcoming studies will require a larger patient cohort, larger ablation volumes, longer follow-up, and careful evaluation of the treatment efficacy using this DISC system. This information could be used to develop targeted interventions aimed at specific subgroups of patients, such as patients with hyperintense uterine fibroids on pretreatment T2w MRI [28] with the use of targeted vessel ablation [48]. Another limitation of our study is that we did the postdischarge follow-up by telephone interviewing. While this may not give us always an objective evaluation (due to absence of nonverbal cues), it provided us with the required information about the safety outcome.

In conclusion, in this proof-of-concept study we successfully performed clinical volumetric MR-HIFU ablation of uterine fibroids using an additional DISC device for reducing the risk of thermal damage to the abdominal wall. On the basis of the small number of patients studied, using the additional DISC device in this trial appears to be safe. No serious (device-related) adverse events occurred.

## Figures and Tables

**Table 1:** Estimated binominal proportion confidence intervals using the Agresti-Coull (adjusted Wald) method [25].

Sample size^a^	Observed outcome		Estimation		
	Successes^a^	Failures^a^	$\hat{p}$	95%-CI	
				Lower	Upper
8	8	0	0.84	0.63	1.00
8	7	1	0.75	0.51	1.00
8	6	2	0.67	0.40	0.94
8	5	3	0.58	0.30	0.87

^a^Values are expressed in numbers; $\hat{p}$: sample proportion of success; CI: confidence interval.

**Table 2:** Baseline characteristics and MRI findings (*n* = 8).

Patient characteristics	
Age (years)^a^	42.5 (38.8–47.8)
BMI (kg/m^2^)^a^	23.1 (19.9–28.1)
Symptoms^b^	
Menorrhagia	3 (38%)
Bulky symptoms	4 (50%)
Infertility	1 (12%)
tSSS^a^	57.9 (38.3–79.0)
Total HRQoL^a^	48.7 (28.9–80.4)

Lesion characteristics	

Number of fibroids^b^	
Solitary fibroid	2 (25%)
Multiple fibroids	6 (75%)
2–5 fibroids	4 (67%)
6–10 fibroids	2 (33%)
Location of fibroids^b^	
Intramural	6 (67%)
Submucosal	3 (33%)
Type of fibroid^b^	
Type 1	4 (45%)
Type 2	3 (33%)
Type 3	2 (22%)
Maximum fibroid diameter (cm)^a^	7.7 (5.1–8.8)
Uterine fibroid volume (cm^3^)^a^	147 (49–338)

^a^Median (interquartile range); ^b^number (percentage); MR-HIFU: MR-guided high-intensity focused ultrasound; tSSS: transformed symptom severity score (range 0–100); high scores indicate more severe symptoms; HRQoL: health-related quality of life (range 0–100); high scores indicate better quality of life; type 1: low signal intensity on T2-weighted imaging; type 2: intermediate signal intensity on T2-weighted imaging; type 3: high signal intensity on T2-weighted imaging; NPV: nonperfused volume.

**Table 3:** Summary of treatment data of each patient treated with the DISC device.

Patient			Patient geometry		Fibroid				Treatment				Patient condition	
ID	Age [years]	BMI [kg/m^2^]	Subcut. fat [mm]	Abdominal scars	Type	Volume [cm^3^]	NPV [cm^3^]	NPV ratio [%]	Energy [kJ]	Time [min]	Energy deposition rate [kJ/h]	Skin temp. [°C]	Skin redness	Subcut. edema
1	43	28.4	18–21	Yes	2	408	249	61	368	223	99	23.0	no	no
2	48	28.1	28–33	Yes	1	25	15	60	169	163	62	21.5	no	no
3	42	22.1	8–14	No	3	268	67	25	331	230	86	22.0	no	no
4	48	24.2	12–14	Yes	2	77	56	72	155	189	49	21.5	no	no
5	47	19.7	8–10	No	1	73	52	71	234	191	74	21.5	no	no
6	38	28.0	14–20	No	1 + 1	17 + 147	14 + 66	82 + 45	240	225	64	20.5	no	no
7	31	20.3	7–15	No	3	200	0	0	294	177	100	21.0	no	no
8	41	18.4	2–4	No	2	415	115	28	183	193	63	20.5	no	no

ID: identification number; temp.: equilibrium temperature as measured in the water reservoir; subcut.: subcutaneous.
